# Curcumin Induces Neural Differentiation of Human Pluripotent Embryonal Carcinoma Cells through the Activation of Autophagy

**DOI:** 10.1155/2019/4378710

**Published:** 2019-01-21

**Authors:** Nudjanad Heebkaew, Narawadee Rujanapun, Phongsakorn Kunhorm, Thiranut Jaroonwitchawan, Nipha Chaicharoenaudomrung, Wilasinee Promjantuek, Parinya Noisa

**Affiliations:** Laboratory of Cell-Based Assays and Innovations, School of Biotechnology, Institute of Agricultural Technology, Suranaree University of Technology, 111 University Avenue, Nakhon Ratchasima 30000, Thailand

## Abstract

Curcumin is a natural polyphenolic compound, isolated from Curcuma longa, and is an important ingredient of Asian foods. Curcumin has revealed its strong activities of anti-inflammatory, antioxidant, and anticancer. The efficient amount of curcumin could induce differentiation of stem cells and promoted the differentiation of glioma-initiating cells; however, the mechanisms underlying neural induction of curcumin have not yet been revealed. In this study, neural-inducing ability of curcumin was explored by using human pluripotent embryonal carcinoma cells, NTERA2 cells. The cells were induced toward neural lineage with curcumin and were compared with a standard neutralizing agent (retinoic acid). It was found that, after 14 days of the induction by curcumin, NTERA2 cells showed neuronal morphology and expressed neural-specific genes, including* NeuroD*,* TUJ1*, and* PAX6*. Importantly, curcumin activated neurogenesis of NTERA2 cells via the activation of autophagy, since autophagy-related genes, such as* LC3*,* LAMP1*, and* ATG5*, were upregulated along with the expression of neural genes. The inhibition of autophagy by chloroquine suppressed both autophagy and neural differentiation, highlighting the positive role of autophagy during neural differentiation. This autophagy-mediated neural differentiation of curcumin was found to be an ROS-dependent manner; curcumin induced ROS generation and suppressed antioxidant gene expression. Altogether, this study proposed the neural-inducing activity of curcumin via the regulation of autophagy within NTERA2 cells and underscored the health beneficial effects of curcumin for neurodegenerative disorders, such as Alzheimer's disease and Parkinson's disease.

## 1. Introduction

The embryonal carcinoma stem cells, named NTERA2 cells, are derived from a human testicular cancer and are able to differentiate into functional postmitotic neurons and other cell types of neural lineages [1–3]. NTERA2 cells exhibit comparable differentiation potential with human embryonic stem cells and could be used to study the early stages of human neurogenesis [4]. In the previous studies, NTERA2 cells were differentiated into neurons, which resembled the vertebrate neurogenesis [5–8]. Besides, the differentiated NTERA2 cells also expressed a variety of neurotransmitters [9, 10] and activated electrical synapses [11, 12]. In addition, NTERA2 cells were found to transform into neurons when exposed to retinoic acid via asymmetric cell division. The differentiated cells loosed the expression of SSEA-3, the pluripotent marker, and were capable of forming the interconnected axon networks and expressed tetanus toxin receptors and neurofilament proteins [1]. The morphological characteristics of neurons could be observed after 10-14 days of the exposure of NTERA2 to retinoic acid. Besides, oligodendrocyte lineage, such as NG2 and GalC-positive cells, could also be generated from the differentiation of NTERA-2 cells [13]. Therefore, NTERA2 cells are considered as the feasible model for studying mechanisms of neural differentiation of the nervous system [6].

Autophagy is a dynamic process of protein degradation within cells and functions as a survival mechanism for homeostasis regulation [14]. Autophagy could also promote cell differentiation by recycling cellular components as well as induction of nonapoptotic cell death [15]. Human myeloid leukemia cell lines (K562 cells) were reported to exhibit high autophagy activity during the* in vitro* differentiation toward neuronal cells [16]. Autophagy activity could be suppressed by the mammalian target of rapamycin (mTOR), an atypical serine/threonine protein kinase, and the expression of mTOR gene was decreased in neuronal mouse neuroblastoma differentiation [17, 18]. Such information supported the involvement of autophagy in neural differentiation, and the ability to control autophagy should improve the generation of neural cells.

Curcumin (diferuloylmethane) is a phytopolyphenol compound isolated from the flowering plant,* Curcuma longa*, commonly known as turmeric. Curcumin possesses a variety of pharmacologic properties, such as anti-inflammatory, antioxidant, and anticancer [19]. Autophagy was reported to be affected by curcumin. Curcumin enhanced the expression of* LC3-I/II*, autophagosomal markers, when it was applied to colon cancer HCT116 cells [20]. Curcumin presented the neuroprotective effects in diseased animal models via the inhibition of A*β* generation. This outcome was a consequence of the induction of autophagy by downregulating PI3K/Akt/mTOR signaling pathway [21]. Previous studies presented that curcumin exhibited the biphasic effects on the proliferation and differentiation of stem cells, including spinal cord neural progenitor cells, embryonic neural progenitor cells, and 3T3-L1 preadipocytes [22–24]. To verify the optimal curcumin concentration as well as the administration time for stem cell differentiation with curcumin, further studies are necessary. Noteworthy, the mechanisms underlying stem cell differentiation of curcumin should also be addressed for a better understanding of curcumin biology. Therefore, the key aim of this current study was to investigate the impact of curcumin on human pluripotent NTERA2 cell differentiation and explore the possible mechanisms of curcumin in mediating of such cell differentiation.

## 2. Materials and Methods

### 2.1. Cell Culture

NTERA2 cells and SH-SY5Y cells were maintained in high-glucose DMEM medium, supplemented with 10% heat-inactivated fetal bovine serum (FBS) and 1% penicillin-streptomycin and glutamine in a humidified incubator containing 5% CO_2_ in air at 37°C. Undifferentiated NTERA2 cells were used as a negative control cell, while SH-SY5Y cells were used in this study as a positive control of standard neuronal cell types. Curcumin and chloroquine (both from Sigma-Aldrich, USA) were dissolved in dimethyl sulfoxide (DMSO) to prepare a stock solution of 100 mM and 10 mg/mL, respectively. Aliquots were stored at 20°C until ready to use and freshly diluted for each experiment. The concentration of DMSO was less than 0.1% in all experiments. For differentiating of NTERA2 cells, the cells were cultured in DMEM medium supplemented with 10% fetal bovine serum (FBS), L-glutamine, non-amino essential, penicillin-streptomycin, and small molecules. Small molecule used in this study to induce neural cell fate of human pluripotent NTERA2 cells was 10 *μ*M retinoic acid, 1 *μ*M curcumin, or 5*μ*M curcumin. Cells were incubated at 37°C in 5% (v/v) CO_2_ for 14 days prior to further characterization.

### 2.2. Measurement of Cell Viability

The preparation of NTERA2 cells and the evaluation of small molecules were performed in 96-well plates containing a final volume of 100 *μ*l/well. Cells were seeded and incubated for desired period of exposure. Then, 10 *μ*l MTT solution was added to each well to achieve a final concentration of 0.45 mg/ml before further incubation for 1 to 4 hours at 37°C in the dark. The media were removed, and the formazan crystal was solubilized in DMSO. The absorbance was measured at 570 nm, using a microplate reader (BMG Labtech, Ortenberg, Germany). The OD 570 nm in control cells will be taken as 100% viability.

### 2.3. Gene Expression

Gene expression was examined by RT-PCR. NTERA2 cells and SH-SY5Y cells were primarily maintained as an adherent culture and were transferred into the T25 flasks at 90% confluence. The cells were then treated with either 10 *μ*M retinoic acid, 1*μ*M curcumin, or 5 *μ*M curcumin for 14 days prior to further characterization. Total RNA was extracted with Macherey-Nagel Kit (Macherey-Nagel, Düeren Germany). Total RNA was then converted to cDNA using TOYOBO kits. The expression of neuron and autophagy genes was performed by PCR (BioRad, USA) using the primer shown in Table 1.

### 2.4. Immunofluorescence

NTERA2 cells and SH-SY5Y cells were primarily maintained as an adherent culture and were transferred into the 24-well plates (sterilized cover slip) at 70% confluence. The cells were then treated with either 10 *μ*M retinoic acid, 1*μ*M curcumin, or 5 *μ*M curcumin for 14 days prior to further characterization. After 14 days, cells were fixed with 4% paraformaldehyde for 30 minutes at room temperature. Cells were washed with PBS twice and permeabilized with 3% bovine serum albumin (BSA) in 0.1% triton-X 100 of PBS for 20 minutes at 4°C. After 20 minutes, cells were incubated with primary antibody: TUJ1 (1:1000) and LC3 (1:1000). All incubations were performed for overnight, and then the samples were incubated with the secondary antibody for 30 minutes. Nuclei were stained with DAPI. All samples were observed under the fluorescence microscope (ZOE™ fluorescence cell imager, BioRad, USA).

### 2.5. Monodansylcadaverine (MDC) Assay for Autophagy Detection

Monodansylcadaverine (MDC) is a lysosomotropic compound and is useful for the identification of autophagic vacuoles. NTERA2 cells were primarily maintained as an adherent culture and were transferred into the 24-well plates (with sterilized cover slip) at 70% confluence. The cells were then treated with either 10 *μ*M retinoic acid, 1*μ*M curcumin, or 5 *μ*M curcumin for 14 days prior to further characterization. After 14 days, cells were fixed in 4% paraformaldehyde (PFA) for 30 minutes at 37°C, and then the cells were washed with PBS. Cells were incubated with 50 *μ*M MDC at 37°C for 20 minutes. After 20 minutes, cells were washed with PBS for 3 times; then cells were observed under fluorescent microscope (ZOE™ fluorescence cell imager, BioRad, USA).

### 2.6. ROS Measurement

NTERA2 cells were investigated ROS generation by 2′, 7′- dichlorofluorescein (DCF) assay. Cells were seeded at a density of 5 × 10^4^ cells/ml in 96-black well plate. The cells were then treated with either 10 *μ*M retinoic acid, 1*μ*M curcumin, or 5 *μ*M curcumin for 24 hours. Cells were exposed to 95 mM H_2_O_2_ for 30 minutes before incubated with 10 *μ*M DCF-DA (diluted from a stock solution in dimethyl sulfoxide (DMSO)) for 1 hour prior to the analysis with Varioskan™ LUX multimode microplate reader (Waltham, Massachusetts, USA).

### 2.7. Statistical Analyses

The data values were shown as mean ± SD from at least three independent experiments. The statistic test was analyzed by one-way ANOVA, followed by* Dunnett's* test for multiple comparisons (SPSS version 16.0 software).* P-value (P)* < 0.05 denoted the presence of statistically significant results.

## 3. Results and Discussion

### 3.1. Curcumin Induced NTERA2 Cell Differentiation

**Figure 1:**
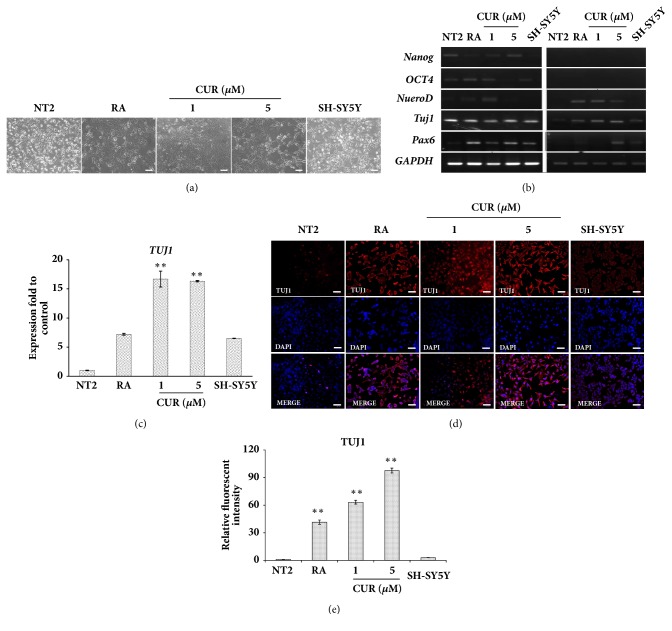
Curcumin induced neural differentiation of NTERA2 cells. (a) The morphology of NTERA2 cells was presented after the treatment of either 10 *μ*M retinoic acid or various concentrations of curcumin for 14 days. The cells were observed by using a phase-contrast microscopy. Scale bar = 100 *μ*m. (b) The expression of* PAX6*,* TUJ1*,* NueroD, OCT4*,* Nanog*, and* GAPDH* was measured by the semi-quantitative RT-PCR before and after the treatment of either 10 *μ*M retinoic acid or different concentrations of curcumin for 7 and 14 days. Undifferentiated NTERA2 (NT2) cells were used as a negative control, while human neuroblastoma SH-SY5Y cells were used as a positive control for the expression of neuronal genes. (c) The relative expression of* TUJ1* was assessed by using* GAPDH* as the internal control. The values were expressed present as mean ± SD, n=3 (^*∗*^*P < 0.05* versus control). (d) The expression of TUJ1 protein was observed by immunofluorescence. Scale bar = 100 *μ*m. (e) The relative fluorescence intensity of TUJ1 protein in differentiated NTERA2 cells was normalized with the undifferentiated control cells. The values were presented as mean ± SD, n=3 (^*∗*^*P < 0.05 *versus control).

**Figure 2:**
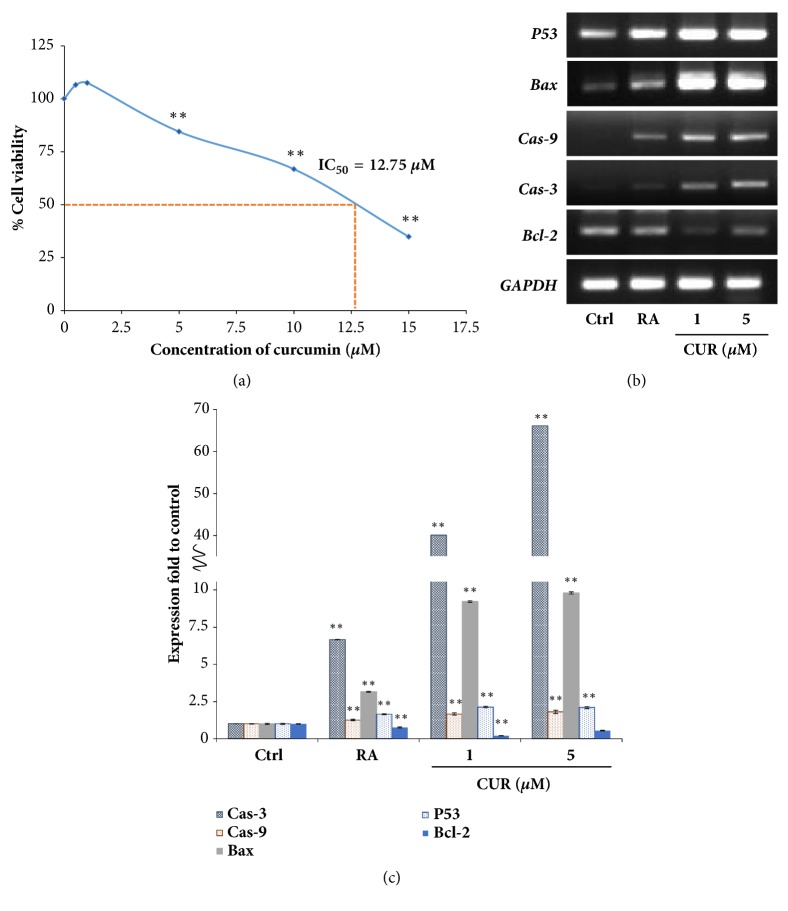
Curcumin induced apoptosis of NTERA2 cells. (a) MTT assay was performed to determine cell viability of NTERA2 cells. The percentage of NTERA2 cell viability after being treated with curcumin at various concentrations was presented, and the IC50 value was 12.75 *μ*M. (b) The expression of apoptosis-related genes,* P53*,* BAX*,* CAS9*,* CAS3*, and* BCL2*, was determined by RT-PCR. (c) The relative expression of apoptosis-related genes was assessed by using GAPDH as the internal control. The values were presented as mean ± SD, n=3 (^*∗*^*P* < 0.05 versus control).

Curcumin possesses multiple biological and pharmacological properties, and neurogenic activity of curcumin became an area of interest [25, 26]. Besides neural cell proliferation [22, 27] and neuroprotection [28, 29], curcumin was also found to increase the rate of neural differentiation from neural stem cells via the activation of the classical WNT pathway [27]. However, the effect of curcumin on promoting neural differentiation of human pluripotent stem cells has not been elucidated. To investigate whether curcumin contained neural-inducing proficiency, human pluripotent NTERA2 cells were chosen as the model in this study. NTERA2 cells are embryonal carcinoma stem cells derived from a human testicular cancer, in which they exhibit pluripotent capacity to differentiate into diverse somatic tissues [30], in particular neural lineage [31]. Hereafter, cell viability assay (Figure 2(a)), NTERA2 cells were supplemented at the subtoxic doses of curcumin (1 and 5 *μ*M) for 14 days. After 14 days of the differentiation, NTERA2 cells presented neuronal morphology with neurofiber and processes in both retinoic acid and curcumin supplemented conditions. The differentiated NTERA2 appeared their similar structure to SH-SY5Y cells at certain degree, which indicated the efficient neural-inducing effects of curcumin (Figure 1(a)). Neuronal identity of curcumin-induced NTERA2 cells was then assessed*, PAX6, NEUROD, and TUJ1* [32], along with the pluripotent genes (*NANOG *and* OCT4* [33]).* NeuroD1*,* TUJ1*, and* PAX6* were highly expressed upon the treatment of curcumin comparing to the undifferentiated control cells (Figure 1(b)). In particular,* TUJ1*, the neuron-specific class III beta-tubulin, was significantly upregulated in both 1 *μ*M curcumin and 5 *μ*M curcumin, and even higher than that of the retinoic acid-treated cells (Figure 1(c)). In the similar manner, the expression of* TUJ1* was found to generally start after 8.5 days of early embryonic development [29] and can be detected throughout the brain development. With respect to adult neurogenesis,* TUJ1* is used as a neuron-specific marker of newly generated cells [31–33] and had been found to label newly generated immature postmitotic neurons [30].* NeuroD* gene was also represented as a transcription factor of later stages of neuronal commitment [34] and used as a neuronal determination genes [35]. Additionally, the upregulated* PAX6* was reflected how such transcription factor mediated cellular processes in curcumin-induced NTERA2 as same as in the precursor cells during embryonic development of the central nervous system which played an important role in the regulation of cell proliferation and neuronal fate determination [36–38]. In contrast, the downregulation of pluripotent genes indicated the loss of differentiation potential of NTERA2 cells after their fate as neuron was determined.

Immunofluorescence staining of TUJ1 confirmed the effect of curcumin on neural differentiation of NTERA2 cells. The red fluorescent signal, representing TUJ1-positive cells, was significantly higher in curcumin-treated NTERA2 cells, compared to the undifferentiated control cells (Figures 1(d) and 1(e)). Such evidence supported that the both effective concentrations of curcumin could induce neural differentiation human embryonal carcinoma cell translationally. Number studies proposed the mechanism of action of curcumin in neurogenesis involving multiple signaling pathways, including WNT/*β*-catenin pathway, ERK/MAPK pathway [26, 39], and WNT signaling pathway [27]. However, the mechanisms behind the differentiation induction were not elucidated and need to be explored in our further experiments.

*NeuroD*,* TUJ1*, and* PAX6* were selected for monitoring neural differentiation of NTERA2 cells in this study because they are well known key determinant factors of neural cell fate.* TUJ1* is presented in newly generated immature neurons and some differentiated neurons [32], while* PAX6* is a human neuroectoderm cell fate determinant and highly expressed in neural progenitor/stem cells [34].* NeuroD1* induces terminal neuronal differentiation. This gene was not expressed in mitotic neural precursor cells, but in cells that undergo neural differentiation [35, 36]. The discrepancy of neural marker expression patterns could be due to the distinct stages of differentiated cells induced by 1 and 5 *μ*M curcumin. The high expression of* NeuroD* and* TUJ1* in 1 *μ*M curcumin treatment reflected the mature neurons derived from NTERA2 cells, while 5 *μ*M curcumin induced high* TUJ1* and* PAX6* expression, referring to neural progenitor/stem cells. The diversity of curcumin-induced neurons signified NTERA2 cells as a resource for neural derivative production.

### 3.2. Curcumin-Induced Neural Cell Differentiation of NTERA2 Involved Apoptosis

Generally, apoptosis, a form of programmed cell death, is one of the cellular mechanisms that occurs during the normal development of the mammalian nervous system and has been observed in populations of developing neural precursor cells, differentiated postmitotic neurons, and glial cells [37, 38, 40]. Such cellular mechanism was stated to be crucial for the establishment of neuronal and glial populations in the correct size to match the number of the innervating neurons with the size of the final target [41]. Plus, it was demonstrated that apoptosis and differentiation were physiological features like chromatin condensation or the need of caspase activity [42]. Prior to any of our experiments, the effect of curcumin on cell viability of NTERA2 were initially investigated by MTT assays (Figure 2(a)). Cells viability was found to reduce at high concentration of curcumin with the IC50 = 12.75 *μ*M. The subtoxic doses of curcumin (1 *μ*M to 5 *μ*M) not only induced neural differentiation of NTERA2 cells, but also triggered apoptosis machinery (Figure 2(b)). The treatment of curcumin was found to increase expression of* P53, BAX CAS9*, and* CAS3*. Cell differentiation and apoptosis are the two-adhered and accompanying processes. For example, the activation of caspase 3 instructed the phosphorylation of protein kinase p38, which consequently activated the neural differentiation transcription factors [43]. Nevertheless, curcumin significantly decreased the expression of antiapoptotic gene,* BCL2 *(Figure 2(c)). Curcumin could also trigger caspase 3-mediated cell death via the activation* GADD153*, which in turn acted as an activator of apoptosis [44]. Thus, this result indicated that the induction of neural differentiation by curcumin was related to the apoptosis pathway in order to maintain the optimal patterns and homeostasis of neurogenesis.

### 3.3. Curcumin Activated Autophagy in NTERA2 Cells

**Figure 3:**
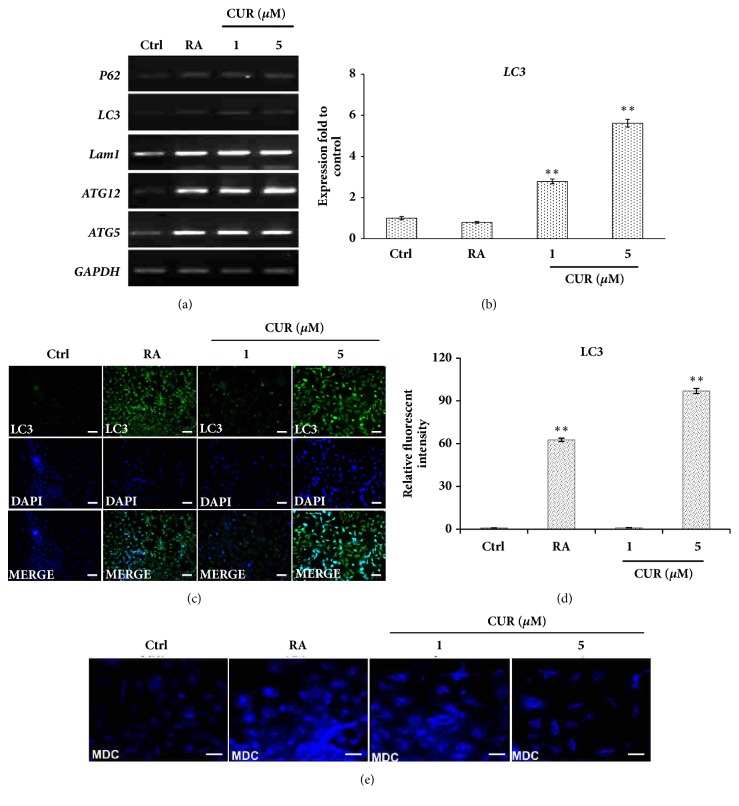
Curcumin activated autophagy during neural differentiation of NTERA2 cells. (a) The mRNA expression levels of autophagy-related genes,* LC3*,* LAMP1*,* ATG5*, and* ATG12*, were quantified by RT-PCR. (b) The relative expression of* LC3* was assessed by using* GAPDH* as the internal control. The values were expressed present as mean ± SD, n=3 (^∗^*P < 0.05* versus control). Scale bar = 100 *μ*m. (c) Immunofluorescence of LC3 protein was performed in NTERA2 cells treated with either 10 *μ*M retinoic acid, 1*μ*M curcumin, or 5*μ*M curcumin for 14 days. (d) The relative fluorescence intensity of LC3 protein in differentiated NTERA2 cells was normalized with the undifferentiated control cells. The values were presented as mean ± SD, n=3 (^*∗*^*P < 0.05 *versus control). (e) MDC assays presented the level of autophagy activity of the differentiated cells at day 14, compared with the undifferentiated control cells.

Autophagy is a lysosome-dependent degradation pathway that involved protein degradation and recycling of cellular component [45]. The significance of autophagy was previously discussed in terms of its positive roles in promoting pluripotent stem cell generation and differentiation [46, 47]. To reveal whether curcumin could trigger neural differentiation through the activation of autophagy, NTERA2 cells were treated with curcumin for 14 days prior to the assessment of autophagy-related genes, including* ATG5*,* ATG12*,* LAM1*, and* LC3 *by RT-PCR. The induced neurons by curcumin evidently surged the expression of autophagy-related genes, and the expression of those genes, in particular* LC3*, exhibited as a dose-dependent manner of curcumin (Figures 3(a) and 3(b)). Immunofluorescence confirmed the activation of autophagy machinery, LC3, when NTERA2 cells were differentiated by retinoic acid and curcumin (Figures 3(c) and 3(d)). NTERA2 cells, treated with either 10 *μ*M retinoic acid or 5 *μ*M curcumin, presented clear fluorescence dots of LC3 staining, while the untreated control cells and 1 *μ*M curcumin-treated cells did not show LC3 staining. To ensure the activation of autophagy, dansylcadaverine (MDC) assay was performed. MDC preferentially accumulates in autophagic vacuoles due to the combination of ion trapping and specific interaction with membrane lipids, causing MDC to be a useful probe for monitoring autophagy [48]. The effect of retinoic acid and curcumin on autophagy activation was tested with NTERA2 cells soon after 24 hours of the differentiation commencement. The cells treated with retinoic acid and curcumin appeared brighter fluorescence aggregates than that of the undifferentiated control cells, indicating the high autophagic activity of the differentiated cells (Figure 3(e)). The activity of autophagy was highlighted here to be a positive regulator during stem cell differentiation.

Several studies found that, in both mouse and human, autophagy was activated during cell differentiation as observed via LC3-II formation on the Western blot and electron microscope. The levels of LC3-II have been correlated with the extent of autophagosome formation. The detection of LC3 expression is a specific and simple technique for monitoring autophagy activity. The expression of LC3-II increased in human glioblastoma cells, U87-MG cells, and U373-MG cells, treated with curcumin in dose- and time-dependent manners. Collectively, these results indicated that curcumin induces autophagy in human brain cancer cells [49]. In addition, human neuroblastoma cells treated with H_2_O_2_ resulted in a time-dependent conversion of LC3 protein from LC3-I to LC3-II and enhanced the activity of autophagy [50]. Thus, it concluded that the increase of LC3-II level was correlated with the elevated autophagy activity.

### 3.4. Inhibition of Autophagy Prevented Neural Differentiation of NTERA2 Cells

**Figure 4:**
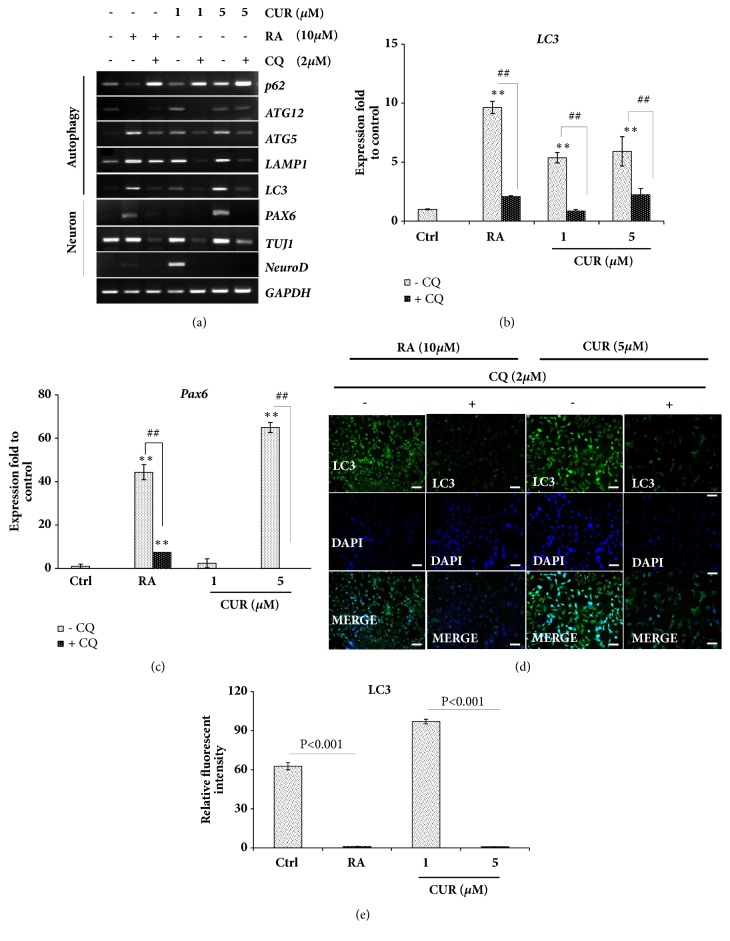
The inhibition of autophagy suppressed neural differentiation of NTERA2 cells. (a) The mRNA expression levels of autophagy-related genes and neural lineag genes were quantified by RT-PCR. Chloroquine, an autophagy inhibitor, was supplemented in order to assess the impact of autophagy during neural differentiation. (b, c) The expression of* LC3* and* PAX6 *was assessed by using* GAPDH* as the internal control. The values were presented as mean ± SD, n=3 (^*∗*^*P < 0.05* versus control). (d) Immunofluorescence of LC3 protein was performed to indicate autophagy activation. Scale bar = 100 *μ*m. (e) The relative fluorescence intensity of LC3 protein in differentiated NTERA2 cells was normalized with the undifferentiated control cells. The values were presented as mean ± SD, n=3 (^*∗*^*P < 0.05* versus control).

Chloroquine, an autophagy inhibitor, was implemented to prove the role of autophagy during curcumin-induced neural differentiation of NTERA2 cells. It was found that the treatment of chloroquine enhanced* P62* expression, a negative regulator of autophagy, while it suppressed autophagy machinery genes, including* LC3*,* LAMP1*,* ATG5*, and* ATG12 *(Figures 4(a) and 4(b)). The neural-inducing activity of both retinoic acid and curcumin was also inhibited by chloroquine, shown by the downregulation of* PAX6*,* TUJ1,* and* NeuroD* in chloroquine-treated cells (Figures 4(a) and 4(c)). To confirm whether neural differentiation was triggered by autophagy, immunofluorescence staining of LC3 protein was performed in cells with or without chloroquine. When chloroquine was absent, the neuron-derived NTERA2 cells with both 10 *μ*M retinoic acid and 5 *μ*M curcumin-treated cells were positively stained for LC3 protein (Figures 4(d) and 4(e)). This was different when chloroquine was applied; the fluorescence intensity of LC3 protein staining was significantly diminished (Figures 4(d) and 4(e)). This was in line with the previous report, demonstrating that the inhibition of autophagy of preadipocytes by* ATG5 *or* ATG7 *defective, or 3-methyladenine autophagy inhibitor (3MA) decreased adipocyte differentiation [51].

### 3.5. Curcumin Induced Autophagy via an ROS-Dependent Manner

**Figure 5:**
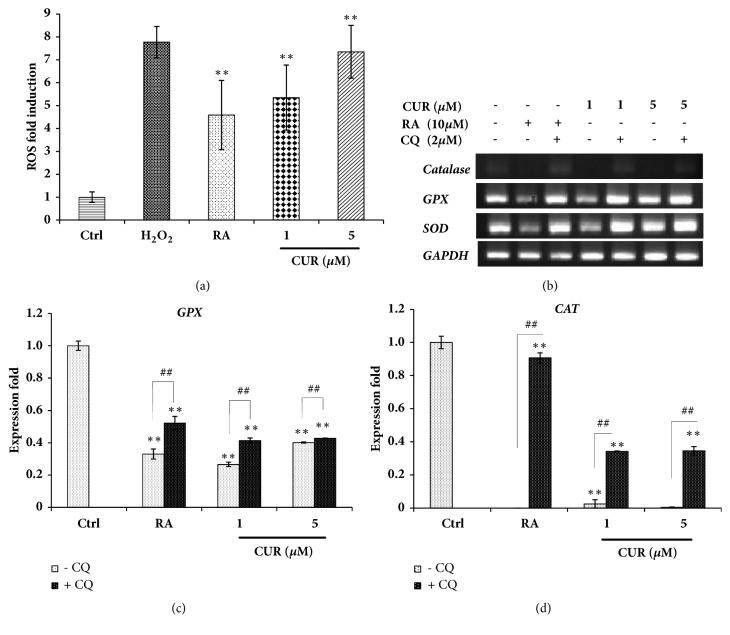
Curcumin induced autophagy via the ROS-dependent manner. (a) The generation of ROS within NTERA2 cells was measured by DCF detection assay. The three independent experiments were performed, and the values were presented as mean ± SD, n=3 (^*∗*^*P < 0.05 *versus control). (b, c, d) The expression of antioxidant genes was assessed by semi- RT-PCR, and* GAPDH* was used as the internal control. Chloroquine was supplemented to verify the effect of autophagy on ROS generation during neural differentiation of NTERA2 cells. The values were presented as mean ± SD, n=3 (^*∗*^*P < 0.05* versus control).

Reactive oxygen species (ROS) are small and highly reactive molecules that can oxidize proteins, lipids, and DNA. ROS, like other free radicals, could serve as signaling molecules in a variety of cellular processes, including cell growth, differentiation, and adhesion [52]. To examine whether ROS interacted with autophagy during NTERA2 cell differentiation, DCF assays were performed. Upon the differentiation, NTERA2 cells induced a significant level of ROS, and the treatment of 5 *μ*M curcumin upsurged the highest production level of ROS (Figure 5(a)). This was in contrast with the expression of antioxidant genes, including* GPX, SOD,* and* CATALASE*, in which they were downregulated in the differentiated NTERA2 cells (Figure 5(b)). The supplementation of chloroquine could recover the expression levels of antioxidant genes, indicating that autophagy prevented ROS production within cells by modulating the level of antioxidant genes (Figures 5(b) and 5(c)). When tightly controlled, ROS serves as signaling molecules by modulating the activity of the oxidized targets. Intracellular ROS functions as signaling molecules to initiate downstream events in regulating the cell cycle, cell differentiation, and apoptosis [53]. Number of evidences proposed an essential role of ROS in the activation of autophagy [54]. In accordance with our finding, curcumin induced ROS production within NTERA2 cells and reduced the expression of antioxidant genes, leading to the activation of autophagy and the induction of neural differentiation of NTERA2 cells. This study and others indicated that neurogenesis required the generation of optimally high levels of ROS, and curcumin could deliver this activity [55]. Interestingly, although the subtoxic doses of curcumin surged ROS in NTERA2-derive neural cells at the comparable level with H_2_O_2_ treatment, substantial number of cell deaths was not observed in this study. This could be due to the biphasic functions of curcumin in the differentiation culture: ROS induction and autophagy activation [56]. Altogether, it was suggested that curcumin was an effective and potent compound to promote neurogenesis in human systems.

## 4. Conclusion

**Figure 6:**
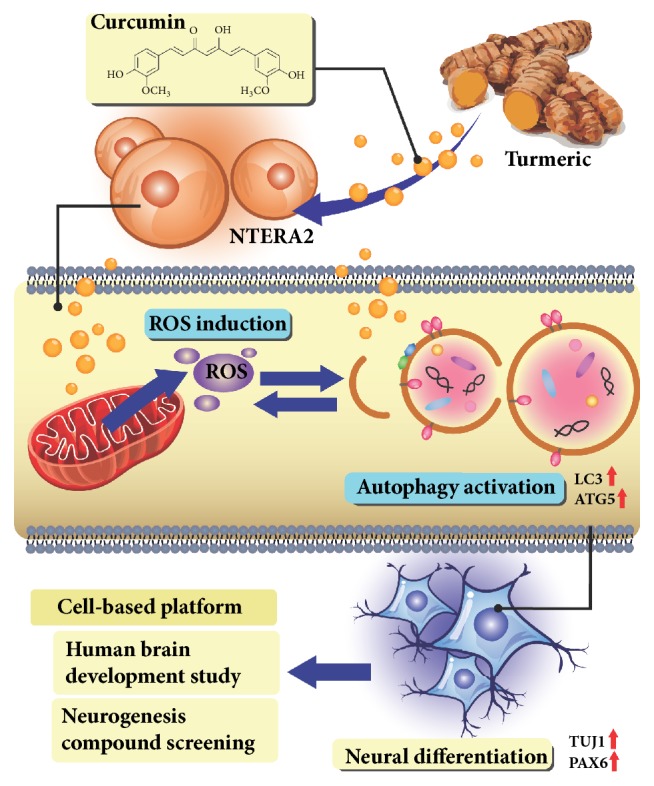
Schematic illustration of the effects of curcumin on neural differentiation. Curcumin induced NTERA2 cells toward neuronal cell fate by activating autophagy in an ROS-dependent manner. This emphasized the feasibility of using NTERA2 cells as a cell-based platform for human brain development study and neurogenesis compound screening.

Curcumin has the advantages of being safe, nontoxic, cheap, and convenient, and it presented here as a potent neural-inducing compound. Curcumin could induce neurogenesis of human pluripotent NTERA2 cells, via the activation of autophagy in an ROS-dependent manner (Figure 6). This study verified the health-beneficial effects of curcumin to be used as a daily food ingredient and could potentially prevent neurological disorders, such as Alzheimer's disease.

## Figures and Tables

**Table 1:** The list of primer sets for PCR reaction of pluripotent genes, neurogenesis genes, apoptosis genes, autophagy-related genes, and ROS genes used in this study.

	**Primer**	**Sequence (5**′**-3**′**)**

**Pluripotent genes**	*GAPDH *(Forward)	5′ACCTGACCTGCCGTCTAGAA3′
	*GAPDH *(Reverse)	5′TCCACCACCCTGTTGCTGTA3′
	*NANOG* (Forward)	5′AGCCTCTACTCTTCCTACCACC3′
	*NANOG *(Reverse)	5′TCCAAAGCAGCCTCCAAGTC3′
	*OCT4* (Forward)	5′GACAACAATGAAAATCTTCAGGAGA3′
	*OCT4* (Reverse)	5′TTCTGGCGCCGGTTACAGAACCA3′

**Neurogenesis genes**	*NeuroD1* (Forward)	5′AGCCCTCTGACTGATTGCAC3′
	*NeuroD1* (Reverse)	5′GTCTATGGGGATCTCGCAGC3′
	*TUJ1* (Forward)	5′GCTCAGGGGCCTTTGGACATCTCTT3′
	*TUJ1* (Reverse)	5′TTTTCACACTCCTTCCGCACCACATC3′
	*PAX6* (Forward)	5′AACAGACACAGCCCTCACAAACA3′
	*PAX6* (Reverse)	5′CGGGAACTTGAACTGGAACTGAC3′

**Apoptosis genes**	*CAS9 *(Forward)	5′AACAGGCAAGCAGCAAAGTT3′
	*CAS9* (Reverse)	5′TCCATCTGTGCCGTAGACAG3′
	*CAS3* (Forward)	5′TTTGTTTGTGTGCTTCTGAGCC3′
	*CAS3* (Reverse)	5′ATTCTGTTGCCACCTTTCGG3′
	*BCL2* (Forward)	5′CGCATCAGGAAGGCTAGAGT3′
	*BCL2* (Reverse)	5′AGCTTCCAGACATTCGGAGA3′
	*BAX* (Forward)	5′AAGCTGAGCGAGTGTCTCAAGCGC3′
	*BAX* (Reverse)	5′TCCCGCCACAAAGATGGTCACG3′
	*P53* (Forward)	5′CCCCTCCTGGCCCCTGTCATCTTC3′
	*P53* (Reverse)	5′GCAGCGCCTCACAACCTCCGTCAT3′

**Autophagy-related gene**	*ATG5* (Forward)	5′ACGCTGGTAACTGAC AAA G3′
	*ATG5* (Reverse)	5′CACATGACATAA AGTGAGCC3′
	*ATG12* (Forward)	5′GAGACACTCCCATAATGAA3′
	*ATG12* (Reverse)	5′GTAGGACCAGTTTACCATC3′
	*LC3* (Forward)	5′GATGTCCGACTTATTCGAGAGC3′
	*LC3* (Reverse)	5′TTGAGCTGTAAGCGCCTTCTA3′
	*P62* (Forward)	5′GCCATTAGGCAAGCTATGTG3′
	*P62* (Reverse)	5′GGTGCAAGAAGCCATTTAGG3′
	*LAM1*(Forward)	5′TCACCGTCGTGTTGTCCTTC3′
	*LAM1*(Reverse)	5′ACCATCCAGGCGTACCTTTC3′

**ROS genes**	*SOD* (Forward)	5′CTAGCGAGTTATGGCGAC3′
	*SOD* (Reverse)	5′CATTGCCCAAGTCTCCAAC3′
	*GPX* (Forward)	5′CGCCAAGAACGAAGAGATTC3′
	*GPX* (Reverse)	5′CAACATCGTTGCGACACAC3′
	CATALASE (Forward)	5′TCCGGGATCTTTTTAACGCCATTG3′
	CATALASE (Reverse)	5′TCGAGCACGGTAGGGACAGTTCAC3′

## Data Availability

The data used to support the findings of this study are available from the corresponding author upon request.

## References

[1] Andrews P. W. (1984). Retinoic acid induces neuronal differentiation of a cloned human embryonal carcinoma cell line in vitro. *Developmental Biology*.

[2] Pleasure S. J., Page C., Lee V. M.-Y. (1992). Pure, postmitotic, polarized human neurons derived from NTera 2 cells provide a system for expressing exogenous proteins in terminally differentiated neurons. *The Journal of Neuroscience*.

[3] Paquet-Durand F., Bicker G. (2007). Human model neurons in studies of brain cell damage and neural repair. *Current Molecular Medicine*.

[4] Schwartz C. M., Spivak C. E., Baker S. C. (2005). NTera2: A model system to study dopaminergic differentiation of human embryonic stem cells. *Stem Cells and Development*.

[5] Przyborski S. A., Morton I. E., Wood A., Andrews P. W. (2000). Developmental regulation of neurogenesis in the pluripotent human embryonal carcinoma cell line NTERA-2. *European Journal of Neuroscience*.

[6] Przyborski S. A., Smith S., Wood A. (2003). Transcriptional profiling of neuronal differentiation by human embryonal carcinoma stem cells in vitro. *Stem Cells*.

[7] Houldsworth J., Heath S. C., Bosl G. J., Studer L., Chaganti R. S. K. (2002). Expression profiling of lineage differentiation in pluripotential human embryonal carcinoma cells. *Cell Growth & Differentiation*.

[8] Smith B., Treadwell J., Zhang D. (2010). Large-scale expression analysis reveals distinct microrna profiles at different stages of human neurodevelopment. *PLoS ONE*.

[9] Guillemain I., Alonso G., Patey G., Privat A., Chaudieu I. (2000). Human NT2 neurons express a large variety of neurotransmission phenotypes in vitro. *Journal of Comparative Neurology*.

[10] Podrygajlo G., Tegenge M. A., Gierse A. (2009). Cellular phenotypes of human model neurons (NT2) after differentiation in aggregate culture. *Cell and Tissue Research*.

[11] Podrygajlo G., Song Y., Schlesinger F., Krampfl K., Bicker G. (2010). Synaptic currents and transmitter responses in human NT2 neurons differentiated in aggregate culture. *Neuroscience Letters*.

[12] Hartley R. S., Margulis M., Fishman P. S., Lee V. M., Tang C. (1999). Functional synapses are formed between human NTera2 (NT2N, hNT) neurons grown on astrocytes. *Journal of Comparative Neurology*.

[13] Marchal-Victorion S., Deleyrolle L., de Weille J. (2003). The human NTERA2 neural cell line generates neurons on growth under neural stem cell conditions and exhibits characteristics of radial glial cells. *Molecular and Cellular Neuroscience*.

[14] Zhuang W., Li B., Long L., Chen L., Huang Q., Liang Z. (2011). Induction of autophagy promotes differentiation of glioma-initiating cells and their radiosensitivity. *International Journal of Cancer*.

[15] Zhuang W., Long L., Zheng B. (2012). Curcumin promotes differentiation of glioma-initiating cells by inducing autophagy. *Cancer Science*.

[16] Wang Z., Hu W., Zhang J.-L., Wu X.-H., Zhou H.-J. (2012). Dihydroartemisinin induces autophagy and inhibits the growth of iron-loaded human myeloid leukemia K562 cells via ROS toxicity. *FEBS Open Bio*.

[17] Lu Y. (2013). Autophagy activator promotes neuronal differentiation of adult adipose-derived stromal cells. *Neural Regeneration Research*.

[18] Zeng M., Zhou J.-N. (2008). Roles of autophagy and mTOR signaling in neuronal differentiation of mouse neuroblastoma cells. *Cellular Signalling*.

[19] Mujoo K., Nikonoff L. E., Sharin V. G., Bryan N. S., Kots A. Y., Murad F. (2012). Curcumin induces differentiation of embryonic stem cells through possible modulation of nitric oxide-cyclic GMP pathway. *Protein & Cell*.

[20] Kantara C., O'Connell M., Sarkar S., Moya S., Ullrich R., Singh P. (2014). Curcumin promotes autophagic survival of a subset of colon cancer stem cells, which are ablated by DCLK1-siRNA. *Cancer Research*.

[21] Wang C., Zhang X., Teng Z., Zhang T., Li Y. (2014). Downregulation of PI3K/Akt/mTOR signaling pathway in curcumin-induced autophagy in APP/PS1 double transgenic mice. *European Journal of Pharmacology*.

[22] Kim S. J., Son T. G., Park H. R. (2008). Curcumin stimulates proliferation of embryonic neural progenitor cells and neurogenesis in the adult hippocampus. *The Journal of Biological Chemistry*.

[23] Kim J. H. (2011). Curcumin stimulates proliferation, stemness acting signals and migration of 3T3-L1 preadipocytes. *International Journal of Molecular Medicine*.

[24] Buhrmann C., Mobasheri A., Matis U., Shakibaei M. (2010). Curcumin mediated suppression of nuclear factor-*κ*B promotes chondrogenic differentiation of mesenchymal stem cells in a high-density co-culture microenvironment. *Arthritis Research & Therapy*.

[25] Jia N., Sun Q., Su Q., Chen G. (2016). SIRT1-mediated deacetylation of PGC1*α* attributes to the protection of curcumin against glutamate excitotoxicity in cortical neurons. *Biochemical and Biophysical Research Communications*.

[26] Tiwari S. K., Agarwal S., Seth B. (2014). Curcumin-loaded nanoparticles potently induce adult neurogenesis and reverse cognitive deficits in Alzheimer's disease model via canonical Wnt/*β*-catenin pathway. *ACS Nano*.

[27] Chen F., Wang H., Xiang X. (2014). Curcumin increased the differentiation rate of neurons in neural stem cells via wnt signaling in vitro study. *Journal of Surgical Research*.

[28] Guo L., Xing Y., Pan R. (2013). Curcumin protects microglia and primary rat cortical neurons against HIV-1 gp120-mediated inflammation and apoptosis. *PLoS ONE*.

[29] Wang Y., Ju B., Zhang Y. (2017). Protective effect of curcumin against oxidative stress-induced injury in rats with Parkinson’s disease through the Wnt/ *β*-catenin signaling pathway. *Cellular Physiology and Biochemistry*.

[30] Andrews P. W. (1984). Pluripotent embryonal carcinoma clones derived from the human teratocarcinoma cell line Tera-2. Differentiation in vivo and in vitro. *Laboratory Investigation*.

[31] Tegenge M. A., Roloff F., Bicker G. (2011). Rapid differentiation of human embryonal carcinoma stem cells (NT2) into neurons for neurite outgrowth analysis. *Cellular and Molecular Neurobiology*.

[32] von Bohlen Und Halbach O. (2007). Immunohistological markers for staging neurogenesis in adult hippocampus. *Cell and Tissue Research*.

[33] Medvedev S. P., Shevchenko A. I., Mazurok N. A., Zakiian S. M. (2008). OCT4 and NANOG are the key genes in the system of pluripotency maintenance in mammalian cells. *Genetika*.

[34] Wu J. Q., Habegger L., Noisa P. (2010). Dynamic transcriptomes during neural differentiation of human embryonic stem cells revealed by short, long, and paired-end sequencing. *Proceedings of the National Acadamy of Sciences of the United States of America*.

[35] Boutin C., Hardt O., de Chevigny A. (2010). NeuroD1 induces terminal neuronal differentiation in olfactory neurogenesis. *Proceedings of the National Acadamy of Sciences of the United States of America*.

[36] Lee J. E. (1996). Neurod and neurogenesis. *Developmental Neuroscience*.

[37] Oppenheim R. W. (1991). Cell death during development of the nervous system. *Annual Review of Neuroscience*.

[38] Jacobson M. D., Weil M., Raff M. C. (1997). Programmed cell death in animal development. *Cell*.

[39] Liao K. K., Wu M. J., Chen P. Y. (2012). Curcuminoids promote neurite outgrowth in PC12 cells through MAPK/ERK- and PKC-dependent pathways. *Journal of Agricultural and Food Chemistry*.

[40] Buss R. R., Sun W., Oppenheim R. W. (2006). Adaptive roles of programmed cell death during nervous system development. *Annual Review of Neuroscience*.

[41] Kristiansen M., Ham J. (2014). Programmed cell death during neuronal development: the sympathetic neuron model. *Cell Death & Differentiation*.

[42] Lanneau D., de Thonel A., Maurel S., Didelot C., Garrido C. (2007). Apoptosis versus cell differentiation: role of heat shock proteins HSP90, HSP70 and HSP27. *Prion*.

[43] Fernando P., Brunette S., Megeney L. A. (2005). Neural stem cell differentiation is dependent upon endogenous caspase 3 activity. *The FASEB Journal*.

[44] Narayan S. (2004). Curcumin, a multi-functional chemopreventive agent, blocks growth of colon cancer cells by targeting *β*-catenin-mediated transactivation and cell-cell adhesion pathways. *Journal of Molecular Histology*.

[45] Klionsky D. J. (2008). Autophagy revisited: a conversation with Christian de Duve. *Autophagy*.

[46] Vessoni A. T., Muotri A. R., Okamoto O. K. (2012). Autophagy in stem cell maintenance and differentiation. *Stem Cells and Development*.

[47] Sotthibundhu A., Promjuntuek W., Liu M., Shen S., Noisa P. (2018). Roles of autophagy in controlling stem cell identity: a perspective of self-renewal and differentiation. *Cell and Tissue Research*.

[48] Munafó D. B., Colombo M. I. (2001). A novel assay to study autophagy: regulation of autophagosome vacuole size by amino acid deprivation. *Journal of Cell Science*.

[49] Aoki H., Takada Y., Kondo S., Sawaya R., Aggarwal B. B., Kondo Y. (2007). Evidence that curcumin suppresses the growth of malignant gliomas in vitro and in vivo through induction of autophagy: role of akt and extracellular signal-regulated kinase signaling pathways. *Molecular Pharmacology*.

[50] Ashabi G., Ahmadiani A., Abdi A., Abraki S. B., Khodagholi F. (2013). Time course study of A*β* formation and neurite outgrowth disruption in differentiated human neuroblastoma cells exposed to H2O2: Protective role of autophagy. *Toxicology in Vitro*.

[51] Singh R. (2009). Autophagy regulates adipose mass and differentiation in mice. *The Journal of Clinical Investigation*.

[52] Vanden Hoek T. L., Li C., Shao Z., Schumacker P. T., Becker L. B. (1997). Significant levels of oxidants are generated by isolated cardiomyocytes during ischemia prior to reperfusion. *Journal of Molecular and Cellular Cardiology*.

[53] Cort A., Timur M., Ozdemir E., Kucuksayan E., Ozben T. (2012). Synergistic anticancer activity of curcumin and bleomycin: An in vitro study using human malignant testicular germ cells. *Molecular Medicine Reports*.

[54] Scherz-Shouval R., Elazar Z. (2011). Regulation of autophagy by ROS: physiology and pathology. *Trends in Biochemical Sciences*.

[55] Shatrova A. N., Lyublinskaya O. G., Borodkina A. V., Burova E. B. (2015). Oxidative stress-promoted responses in human endometrial stem cells and lung embryonic fibroblasts. *Tsitologiya*.

[56] Jaroonwitchawan T., Chaicharoenaudomrung N., Namkaew J., Noisa P. (2017). Curcumin attenuates paraquat-induced cell death in human neuroblastoma cells through modulating oxidative stress and autophagy. *Neuroscience Letters*.

